# Which is the best femoral implant in children with osteogenesis imperfecta? a retrospective cohort study of 783 procedures

**DOI:** 10.1186/s12891-023-06222-2

**Published:** 2023-02-09

**Authors:** Hongjiang Yang, Bo Li, Cong Xing, Shijie Gao, Wenbiao Zhu, Yang Xiong, Xiuzhi Ren, Guangzhi Ning

**Affiliations:** 1 Department of Orthopedics, Tianjin Medical University General Hospital, 300052 Tianjin, China; 2 Tianjin Key Laboratory of Spine and Spinal Cord Injury, Tianjin Medical University, 300052 Tianjin, China; 3 Department of Pediatric Orthopedics, WuQing People Hospital, 301700 Tianjin, China

**Keywords:** Osteogenesis imperfecta, Telescopic rod, Implant choice, Femoral surgeries

## Abstract

**Background:**

Osteogenesis imperfecta (OI) is a hereditary genetic disorder characterized by bone fragility and extremity deformities. The surgical management for long-bone fractures and deformities in OI remains a challenge. We aimed to compare clinical outcomes after femoral surgery splinted with the telescopic rod, the plate and screws, the elastic nail and the non-elongating rod in setting of OI.

**Methods:**

A retrospective cohort study included 783 femoral procedures (mean age 6.00 (interquartile range (IQR) 5.00) years, 335 (42.8%) females) was conducted, and individuals were categorized into four groups according to implants. After verifying comparability among the groups, revision rate and implant survival period were compared among the Sillence types and the same comparison were made among four groups within each Sillence type. The incidence of refractures, deformities, and implant-related complications were also compared among the four groups.

**Results:**

There were no significant differences in demographic information among the four groups in terms of sex (*p* = 0.101), laterality (*p* = 0.587), Sillence type (*p* = 0.122), and postoperative follow-up period (*p* = 0.214). In total, children with Sillence type III had the highest revision rate and the shortest implant survival period; children with Sillence type I had the lowest revision rate and the longest implant survival period; and children with Sillence type IV had the revision rate and the implant survival period between those observed in Sillence types I and III. In Sillence types III and IV, the telescopic rod had lower revision rate (III 24.8%; IV 20.9%) compared to the plate (III 97.2%, ***p***<0.001; IV 80.3%, *p*<0.001), the elastic nail (III 100.0%, *p*=0.019; IV 73.9%, *p*<0.001) and the non-elongating rod (III 65.0%, *p*<0.001; IV46.9%, *p*<0.001); the median implant survival period of the telescopic rod (III 48.00 (IQR 28.50) months; IV 43.00 (33.00) months) is longer than the plate (III 11.00 (9.00) months, *p*<0.001; IV 19.00 (20.00) months, *p*<0.001), the elastic nail (III 45.00 (37.75) months, *p*=1.000; IV 19.00 (35.00) months, *p*=0.028) and the non-elongating rod (III 39.00 (31.75) months, *p*=0.473; IV 38.50 (29.75) months, *p*=1.000).A similar trend was observed in Sillence type I (*p* = 0.063, *p* = 0.003; respectively). In addition, the incidence of refracture (15.5%), deformity (2.8%) and implant-related complications (23.1%) were also statistically lower in the telescopic rod group.

**Conclusion:**

In our cohort, lower revision rate and longer implant survival period were observed in telescopic rod group. This was mainly due to the significant lower incidence of refracture, deformity and implant-related complications with the use of telescopic rod.

**Supplementary Information:**

The online version contains supplementary material available at 10.1186/s12891-023-06222-2.

## Background

Osteogenesis imperfecta (OI) is a hereditary congenital disorder characterized by bone fragility and extremity deformities [1]. It has been reported that the incidence of OI is 1/15000 ~ 1/20000 and there are more than 100,000 individuals with OI in China [2, 3]. Sillence classification as the classic clinical classification originated in 1979, categorizing OI into four types on the basis of different clinical and radiographic presentations [4]. Almost 90% of patients are caused by gene mutations that encode type I collagen—the major protein component of the extracellular matrix in the bone [5]. The multidisciplinary management strategy has been proposed for this multi-systemic disease, including tailored orthopedic intervention, cyclic anti-osteoporosis medications and positive rehabilitation [6, 7]. Surgical intervention, preventing refractures and deformity by providing structural support for weakened but growing bone, obviously is the key step in the management of children with OI [8]. Therefore, choosing appropriate implants for children with OI has become the focus of surgeons.

In 1963, Robert Bailey introduced the Dubow-Bailey telescopic rod, the anchorage of which consisted of a T-piece in male part and a crimped tip in female part [9]. Although the appearance of the original telescopic rod was more successful at reducing frequent revision due to patient growth, this success was accompanied by a high incidence (50% to 60% rate) of complications [10–13]. Based on the Dubow-Bailey telescopic rod, Sheffield telescopic rod appeared with a larger and fixed T-piece in female part that effectively reduced the risk of proximal migration [14, 15]. Still, positioning the rod via arthrotomy inevitably caused harm to the joint [16, 17]. It was a watershed that Francois Fassier and Pierre Duval designed new telescopic rod with two screw ends with the advantages of avoiding knee or ankle arthrotomies and reducing soft tissue injury [18]. Later, Cho et al. modified the Sheffield telescopic rod by replacing the T-piece anchorage with easily detachable pin, which was called interlocking telescopic rod [19]. However, no long-term data for this device are available, and the risk of rod migration are still high. Before the appearance of the telescopic rod, the implant alternatives were limited to plate, elastic nail and non-elongating rod. But splinting with these implants had not achieved satisfactory outcomes [20–22]. A previous study reported that the combination of rigid plates and the weak bone will result in increased fracture above or below the plate. Besides, bone behind the plate would be further resorbed without any mechanical stress [23]. The non-elongating rod can provide fixation with the entire bone for a short duration, but it will become relatively shorter as long as the bone grows, resulting in the appearance of the bone segments without support by implants. The unsupported bone segment is prone to fracture and deformity, followed by implant protrusion [24]. The elastic nail has a small diameter and is suitable for patients with a thinner medullary cavity, but deformity and refracture frequently occurred because of insufficient strength [20].

Preventing refracture and deformity by providing structural support obviously is the key step in the management of children with OI, but there is a paucity of evidence for the implant choice. This study, with the aim of exploring which femoral implant is more suitable for children with OI, was to compare the clinical outcomes of four implants based on revision rate, implant survival period and incidence of refractures, deformities and implant-related complications.

## Methods

### Study details

**Fig. 1:**
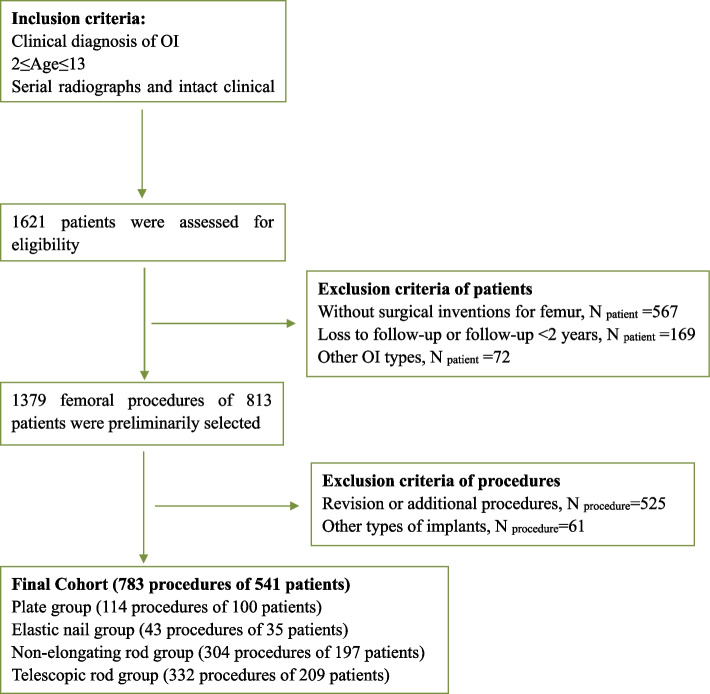
Flow chart on inclusion and exclusion of this cohort study. Other OI types represented some rare types defined by genetic test. And our cohort exclusively include patients with Sillence types I, III and IV. Other types of implants consist of Kirschner wire, intramedullary rod with supplemental plate and screws

This article observed the telescopic rod, the plate, the elastic nail and the non-telescopic rod using an initially surgical controlled, single-surgical team, retrospective design comparing four implants for the surgical management of femur in children with OI. 783 procedures (541 children) with diagnosis of OI were enrolled from 2001 to 2020 in our center. The flow of inclusion and exclusion flowchart was shown in (Fig. 1). To avoid the bias of different outcomes between initial operation and following operation, we exclusively enrolled initial operation of the femur and all procedures were conducted or followed up by our surgical team. The follow-up period was set at a minimum of 24 months and a maximum of 120 months in order to minimize the chronologic bias. By the data extraction was finished, the final cohort comprised 783 femoral procedures (335 females (42.8%) and 448 males (57.2%)), and the overall age range was 2-13 years old (mean age 6.00 (IQR) 5.00) years). Overall, 97 had Sillence type I, 282 had Sillence type III, and 404 had Sillence type IV. 114 procedures (100 patients) comprised the plate and screws group, 43 procedures (35 patients) comprised the elastic nail group, 304 procedures (197 patients) comprised the non-elongating rod group, and 322 procedures (209 patients) comprised the telescopic rod group. The non-elongating rod group consisted of 142 Improved Rush rods, 143 Rush rods and 19 Peter-Wiliams nails. All of telescopic rods were Fassier-Duval telescopic rod. This retrospective study was approved by the Medical Ethical Committee of Tianjin Medical University General Hospital in China. And the retrospective study is anonymous, and the requirement for informed consent was therefore waived by the ethics committee of Tianjin Medical University General Hospital.

### Data collection

The following data were collected: sex, Sillence type (I, III, IV), age of implant insertion, post-operative follow-up period, the revision procedures, implant survival period, and incidence of complications. All data were captured from intact clinical data in our center.

The classification was made basing on the Sillence type: Sillence type I (mild); Sillence type II (perinatally lethal); Sillence type III (severe); and Sillence type IV (moderate). The classification was finished by three surgeons and no significant difference was found. The revision procedures were defined as the replacement or removal of implants owing to unpredictable fractures and progressive deformities. The implant survival period represented the duration from the point of implant insertion (starting point) to the point of implants replacement or removal (end point). Scheduled implant removal, slight displacement of implant and implant bending that had no impact on physical function were not considered an end point. The incidents of refractures, deformities and implant-related complications (including implants migration, breakage or bending of implant, growth attest or nonunion of bone) were documented. It must be said that one femur in each group may have two of these complications.

### Indication for implants

The indication of implants depended on the age of children, the status of femur and the availability of devices. Patients with OI and a femoral fracture or deformity who are older than 2–3 years of age were treated with surgical intervention, and patients younger than 2 years of age were usually treated conservative treatment. The intramedullary rod was used for patients with wide femur canals and those with narrower canals were immobilized with the plate or the elastic nail. The telescopic rods were exclusively used for patients with indications after 2012 when the telescopic system was available in our center.

### Statistical methods

Statistical results were analyzed by using the software SPSS 25 (IBM Corporation, Armonk, New York). Normality of distribution was evaluated using the Shapiro–Wilk tests. Continuous variables (age of implant insertion, follow-up period, survival period of implant) were described as median and interquartile ranges, and were compared among the groups using the Kruskal–Wallis test with Bonferroni’s post comparisons for more than two groups because of the asymmetric distribution of data after the normality test. Categorical variables (gender, laterality, Sillence classification, revision procedures) were expressed by relative frequencies and percentage, and were compared among the groups using the chi-square test followed by Bonferroni’s multiple-comparison tests. The level of significant difference was set at *p* < 0.05.

## Results

### Demographic characteristics

**Fig. 2:**
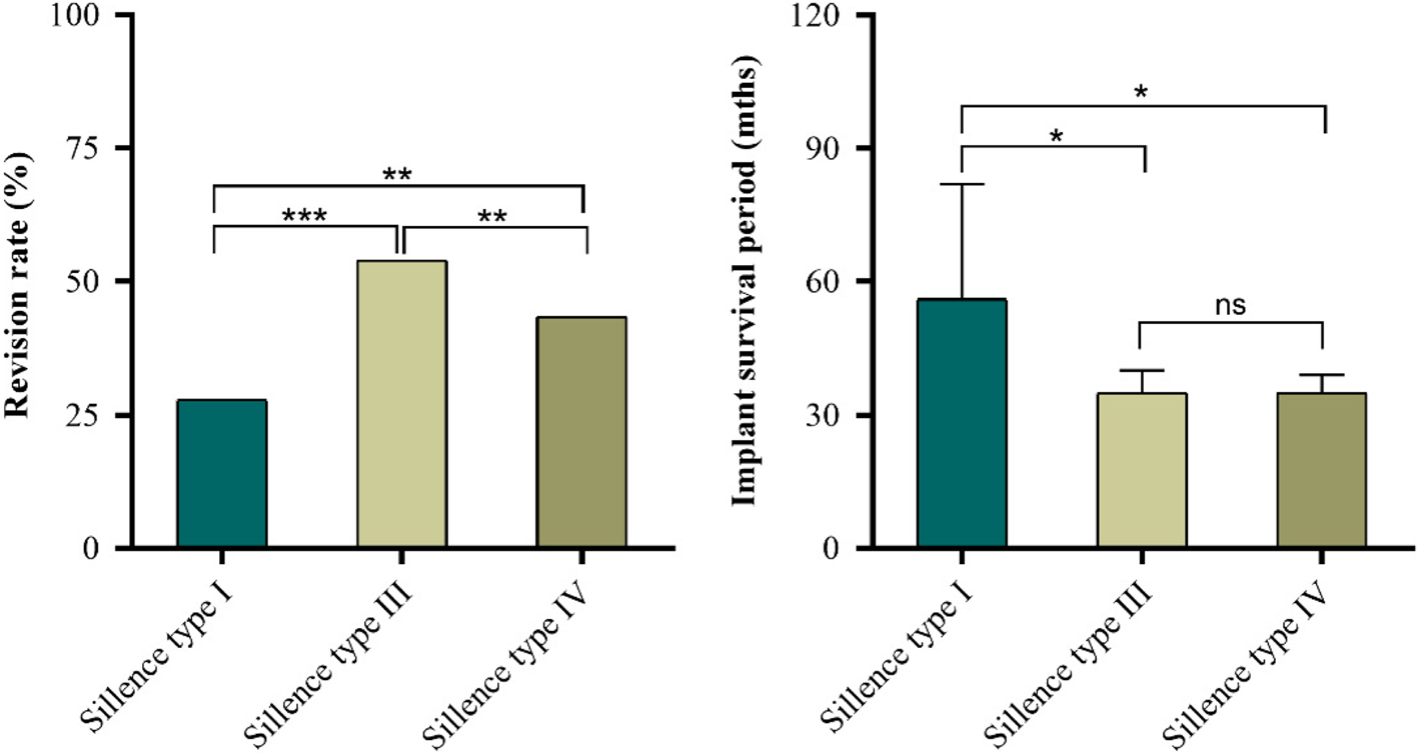
The revision rate and survival period of implants were compared after categorizing by Sillence types. *p < 0.05, **p < 0.01, ***p < 0.001

783 procedures (541 children) were included in the final cohort by December 2020, the inclusion flowchart was detailed in (Fig. 1). The demographic data of four groups were compared (Table. 1) and no significant difference was found among the four groups in reference to gender (*p* = 0.101), laterality (*p* = 0.587), Sillence classification (*p* = 0.122) and postoperative follow-up period (*p* = 0.214). The median age of surgery in telescopic rod group is significantly younger than non-elongating rod group (*p* = 0.003). The median postoperative follow-up period of all participants was 63.00 months (IQR, 36.00). In total, children with Sillence type III (severe) had the highest revision rate (152/282, 53.9%) and the shortest implant survival period (median/range, 35.00/37.00; 95% confidence interval [CI], 33.97–42.04); Children with Sillence type I (mild) had the lowest revision rate (27/97, 27.8%) and the longest implant survival period (56.00/63.00, 43.20–70.20); And children with Sillence type IV (moderate) had the revision rate (175/229, 43.3%) and the implant survival period (35.00/34.00, 35.12–43.16) between those observed in Sillence types I and III (Fig. 2).

**Table 1:** People demographics

Demographics	Plate and screws (*n* = 114)	Elastic nail (*n* = 43)	Non-elongating rod (*n* = 304)	Telescopic rod (*n* = 322)	*p*-value
Sex*, n (%)					0.101^#^
Female	39(34.20)	16(37.20)	143(47.00)	137(42.50)	
Male	75(65.80)	27(62.80)	161(53.00)	185(57.50)	
Laterality*, n (%)					0.587^#^
Left	58(50.90)	17(39.50)	146(48.00)	161(50.00)	
Right	56(49.10)	26(60.50)	158(52.00)	161(50.00)	
Sillence*, n (%)					0.122^#^
Sillence type I	17(14.90)	8(18.60)	25(8.20)	47(14.60)	
Sillence type III	36(31.60)	12(27.90)	117(38.50)	117(36.30)	
Sillence type IV	41(53.50)	23(53.50)	162(53.30)	162(49.10)	
Age of implant insertion, yrs					0.003^^^
Mean (SD)	6.29(3.13)	6.46(3.04)	6.94(3.55)	5.88(3.09)	
Median (IQR)	6.00(4.50)	6.00(4.00)	7.00(6.00) ^a^	5.00(5.00) ^b^	
Postoperative follow-up, mths					0.214^^^
Mean (SD)	67.10(29.52)	71.58(31.95)	68.02(28.31)	62.91(18.08)	
Median (IQR)	60.50(42.50)	71.00(54.00)	66.00(47.75)	62.00(26.00)	

The categorical and continuous variable of four groups were compared, no significant intergroup difference was found in sex, laterality, Sillence and postoperative follow-up (*p* = 0.101, *p* = 0.587, *p* = 0.122, *p* = 0.214; respectively). Except for significant difference between non-elongating rod (^a^) and telescopic rod (^b^) (*p* = 0.003), there was no significant difference in age of implant insertion among groups

^*^The values are expressed as the number of femoral procedures

^#^Pearson's chi-square; ^^^Kruskal–Wallis H test

### Children underwent femoral surgeries splinting with the telescopic rod had lower revision rate and longer implant survival period, especially for Sillence types III and IV

**Fig. 3:**
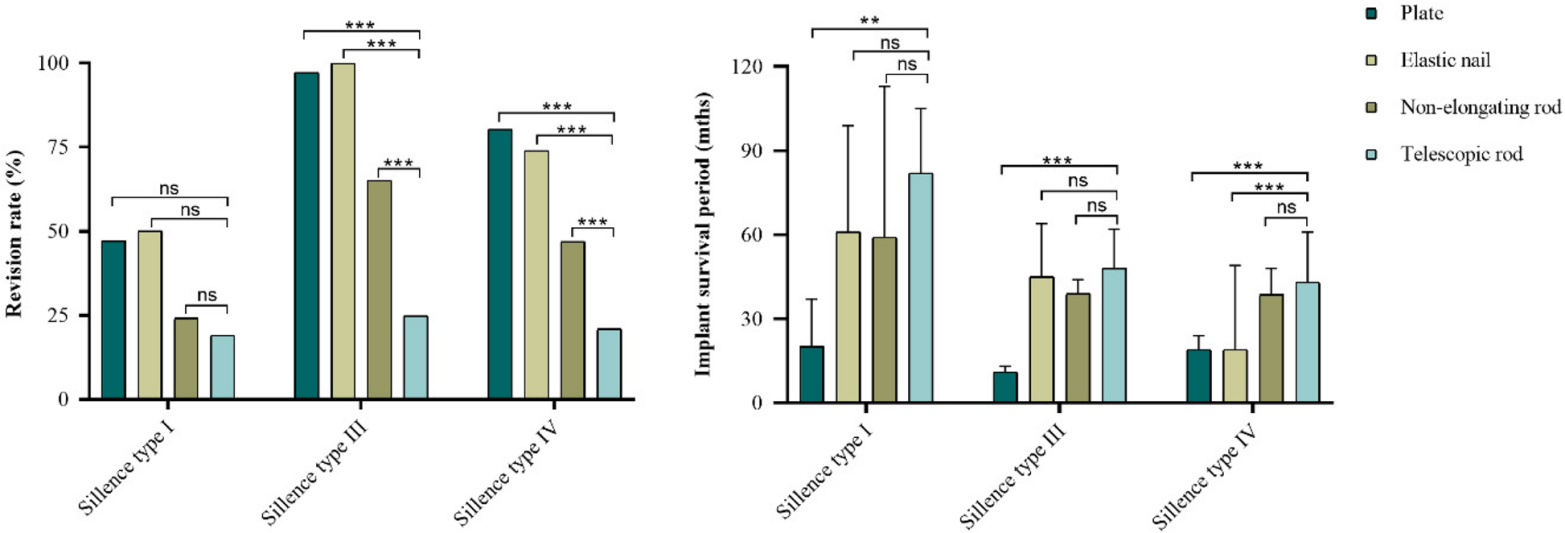
The revision rate and implant survival period of the telescopic rod were compared with plate, elastic nail and non-elongating rod after categorizing by Sillence types. *p < 0.05, **p < 0.01, ***p < 0.001

The revision rate and implant survival period categorized by Sillence type and implant were depicted in (Table. 2) and (Table. 3). For children with Sillence type III, 29 of 117 femurs (24.8%) in telescopic rod group underwent revision procedures versus 35 of 36 femurs (97.2%, *p* < 0.001) in plate group, 12 of 12 femurs (100.0%, *p* < 0.001) in elastic group and 76 of 117 femurs (65.0%, *p* < 0.001) in non-elongating rod group (Fig. 3a); The median implant survival period were 48.00 months (95% CI, 43.54–58.59) in telescopic rod group, 11.00 months (95% CI, 9.91–16.37, *p* < 0.001) in plate group, 45.00 months (95% CI, 29.34–74.48, *p* = 1.000) in elastic nail group, and 39.00 months (95% CI, 37.15–47.39, *p* = 0.473) in non-elongating rod group (Fig. 3b). For children with Sillence type IV, 33 of 158 femurs (20.9%) in telescopic rod group underwent revision procedures versus 49 of 67 femurs (80.3%, *p* < 0.001) in plate group, 17 of 23 femurs (73.9%, *p* < 0.001) in elastic group and 76 of 162 femurs (46.9%, *p* < 0.001) in non-elongating rod group (Fig. 3a); The median implant survival period were 43.00 months (95% CI, 41.91–55.54) in telescopic rod group, 19.00 months (95% CI, 18.18–35.53, *p* < 0.001) in plate group, 19.00 months (95% CI, 18.07–58.98, *p* = 0.028) in elastic nail group, and 38.50 months (95% CI, 38.19–47.88, *p* = 1.000) in non-elongating rod group (Fig. 3b). For children with Sillence type I, there was no difference in revision rate (*p* = 0.063) among the groups, and only the implant survival period of telescopic rod group was longer than plate group (*p* = 0.001) (Fig. 3). The telescopic rod group had lower revision rate and longer implant survival period compared with other three groups in Sillence types III and IV, and a similar trend was found in Sillence type I even if there was no statistical difference.

**Table 2:** Revision rate

	**Plate and screws**	**Elastic nail**	**Non-elongating rod**	**Telescopic rod**	**Total**
Sillence, n/N (%)					
Sillence type I	8/17(47.1)	4/8(50.0)	6/25(24.0)	9/47(19.1)	27/97(27.8)
Sillence type III	35/36(97.2)	12/12(100.0)	76/117(65.0)	29/117(24.8)	152/282(53.9)
Sillence type IV	49/61(80.3)	17/23(73.9)	76/162(46.9)	33/158(20.9)	175/404(43.3)
Total, n/N (%)	92/114(80.7)	33/43(76.7)	158/304(52.0)	71/322(22.0)	354/783(45.2)

The revision rate was compared after categorizing by Sillence types. The comparison results were intuitively presented in Fig. 2 and Fig. 3. n/N means revision procedures/total procedures

**Table 3:** Implant survival period

	**Plate and screws**	**Elastic nail**	**Non-elongating rod**	**Telescopic rod**	**Total**
Sillence, mths					
Sillence type I	20.00(15.50)	61.00(46.00)	59.00(33.00)	82.00(30.50)	56.00(63.00)
Sillence type III	11.00(9.00)	45.00(37.75)	39.00(31.75)	48.00(28.50)	35.00(37.00)
Sillence type IV	19.00(20.00)	19.00(35.00)	38.50(29.75)	43.00(33.00)	35.00(34.00)
Total, mths	14.00(15.00)	35.00(42.50)	39.50(30.25)	53.00(32.00)	16.00(25.00)

The implant survival period was compared after categorizing by Sillence types. The comparison results were intuitively presented in Fig. 2 and Fig. 3. The implant survival period was described as median and interquartile ranges as a result of the asymmetric distribution of data after normality test

### The telescopic rod had lower incidence of refracture, deformity and implant-related complications

**Fig. 4:**
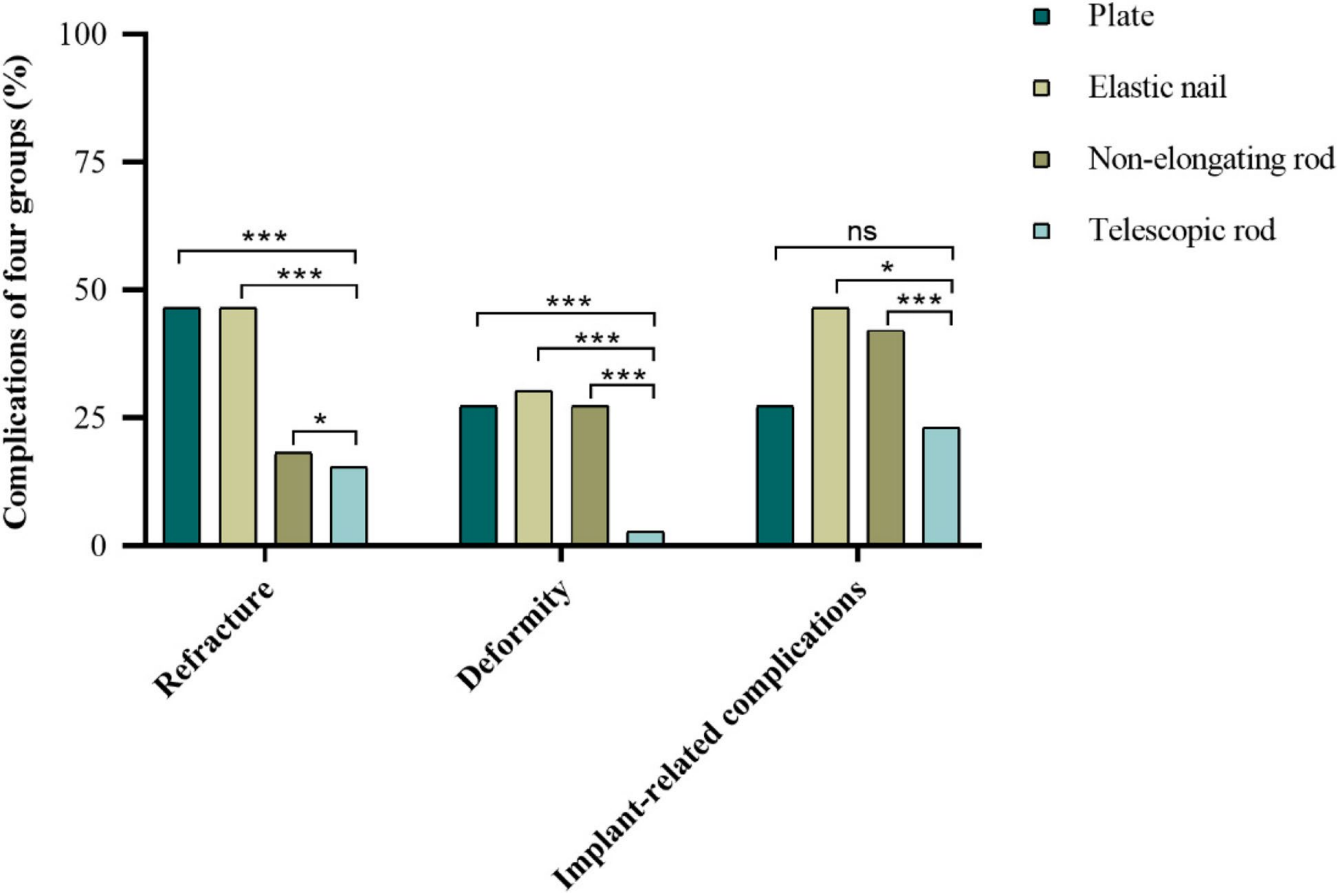
The risk of refracture, deformity and implant-related complications of the telescopic rod were compared with plate, elastic nail and non-elongating rod. *p < 0.05, **p < 0.01, ***p < 0.001

The incidence of refracture, deformity and implant-related complications of the telescopic rod were compared with plate, elastic nail and non-elongating rod (Table. 4). The telescopic rod group had a lower incidence of refractures (50 of 322 femurs, 15.5%) versus (53 of 114 femurs, 46.5%) in plate group, (20 of 43 femurs, 46.5%) in elastic nail group and (55 of 304 femurs, 18.1%) in non-elongating rod group (*p* < 0.001); Besides, the telescopic rod group had a lower incidence of deformities (9 of 322 femurs, 2.8%) versus (31 of 114 femurs, 27.2%) in plate group, (13 of 43 femurs, 30.2%) in elastic nail group, (83 of 304 femurs, 27.3%) in non-elongating rod group (*p* < 0.001); 95 femurs (95 of 322, 23.1%) in the telescopic rod group had implant-related complications compared with 31 (31 of 114, 27.2%) in plate group, 20 (20 of 43, 46.5%) in elastic nail group, 128 (128 of 304, 42.1%) in non-elongating rod group (*p* < 0.001) (Fig. 4). Moreover, the details of implant-related complications were collected (Table. 4), and the most common complication of each implant were shown in (Supplementary Fig 1). The implant-related complications of telescopic rod consisted of breakage or bending of rod (*n* = 38), proximal migration (*n* = 27), distal migration (*n* = 23), growth arrest of the proximal or distal physis (*n* = 5) and nonunion (*n* = 2); 20 femurs in plate group underwent screw pull-out and 11 femurs showed nonunion; There were 13 femurs with nail migration, 4 femurs with breakage or bending of nail and 3 femurs with nonunion in elastic nail group; And there were 56 femurs with rod migration, 47 femurs underwent outgrowing the rod, 17 femurs with breakage or bending of rod and 17 femurs with nonunion in non-elongating rod group. The telescopic rod group had a lower proportion of complications compared with the other three groups.

**Table 4:** Incidence of refracture, deformity and implant-related complications

	Refracture*	Deformity*	Implant-related complications*
Plate and screws (Total = 114)	53(46.5%)	31(27.2%)	31(27.2%) Screw pull-out (*n* = 20) Nonunion (*n* = 11)
Elastic nail (Total = 43)	20(46.5%)	13(30.2%)	20(46.5%) Nail migration (*n* = 13) Breakage or bending of nail (*n* = 4) Nonunion (*n* = 3)
Non-elongating rod (Total = 304)	55(18.1%)	83(27.3%)	128(42.1%) Rod migration (*n* = 56) Bone outgrowing the rod (*n* = 47) Breakage or bending of rod (*n* = 17) Nonunion (*n* = 17)
Telescopic rod (Total = 322)	50(15.5%)	9(2.8%)	95(23.1%) Breakage or bending of rod (*n* = 38) Proximal migration (*n* = 27) Distal migration (*n* = 23) Growth arrest of the proximal or distal physis (*n* = 5) Nonunion (*n* = 2)
*p*-value^#^	*p* < 0.001	*p* < 0.001	*p* < 0.001

The refracture, deformity and implants-related complications of four groups were compared. The complications of telescopic rod were statistically lower than others

^*^The values were expressed as the number of femoral procedures and one femur in each group may have two of these complications

^#^Pearson's chi-square

## Discussion

This study explored the outcomes of four implants for femur in children with OI by comparing the revision rate, survival period of implant and incidence of complications. We found lower revision rate and longer implant survival period in telescopic rod group, especially for Sillence types III and IV. This is mainly due to lower risk of refractures, deformities and implant-related complications in telescopic rod group compared with other three implants.

In our cohort, lower revision rate and longer implant survival period were found in telescopic rod group for Sillence types III and IV during 53-month median follow-up. Consistent with our study, Bailey and Dubow contrived a telescoping rod system with a significant decrease in revision rate from 24%–51% to 12%–27% [25]. The telescopic rod elongated with the growth of bone, contributing to lower revision rate, but this system also had many complications frequently relative with the T-piece [10, 11, 16]. The appearance of FD telescopic rod addressed this issue with a screw-like fixation resulting in a further decrease in revision rate. Spahn et al. reported that eightfold fewer revision procedures and twofold longer implant survival period than with non-elongating rod [24]. Telescopic rod has obvious advantages in reducing the revision surgeries by providing persistent support for long-bone in setting of OI. Historically, plate and screws, elastic nail and non-elongating rod (Rush, Improved Rush, and Peter-Williams devices) were used for fracture stabilization in children with OI. However, Fixation with plate and screws will create stress concentrations above or below the plate, leading to increasing risk of fracture around plate edges. Enright WJ et al. discouraged the use of plates in Sillence III for long bones [21]. The elastic nail had high incidence of refracture and deformity, Popkov et al. recently confirmed that elastic nail with high revision rate shouldn’t be used in patients with OI [26]. Although the static rod can provide fixation with the entire bone for a short duration, but it had an obvious drawback that the static rod became shorter with the growth of bones [27, 28]. A meta-analysis of 7 studies (*n* = 229 patients) reported a 39.4% reoperation rate with the use of non-elongating rod [22]. In our cohort, higher revision rate, shorter implant survival period and high incidence of complications also were found in the plate, the elastic and the non-elongating rod groups. Therefore, the telescopic rod should be given priority to the surgical management, especially in children with Sillence types III and IV.

The telescopic rod had lower post-operative incidence of refracture, deformity and implant-related complications compared with the plate, elastic nail and non-elongating rod groups. Although revision surgery was mainly related to subsequent fracture and growth deformity, complications related to the anchorage of the telescopic system can’t be neglected [29–31]. In telescopic group, there were 27 femurs with proximal migration and 23 femurs with distal migration, all of which (15.5%) can be contributed to the anchorage of telescopic system. Azzam et al. reported 9 rod migrations of Fassier-Duval rod in 58 patients with a mean follow-up of 5 years [32]. In another cohort with 1.6 year-median follow-up, migration of the sleeve occurred in one of eight patients [33]. Recently, studies reported that fewer complications related to anchorage system with the use of interlocking telescopic rod [34, 35]. It indicated that the complications can be minimized further with more effort to improve the anchorage of telescopic system.

The age of implant insertion in the telescopic rod group was statistically younger than non-elongating rod group in this study, because the telescopic rod was preferably used for younger children with dynamic and growing bone. Besides that, as a retrospective cohort spanned 20 years, the lower revision rate and lower incidence of complications in telescopic rod group may be contributed to better surgical management and anti-osteoporosis therapy. In addition, bilateral femurs from a single patient were considered independently in the statistical analysis. Despite these limitations, this review of our experience helped us optimize our surgical management in setting of OI.

In conclusion, the results showed that a lower revision rate and longer survival period in the telescopic rod group, especially in the types III and IV OI. This is mainly due to the lower incidence of refracture, deformity and implant-related complications with the use of telescopic rod. The results of this study may be generalizable because of the impressive number of patients and long follow-up period, which provided evidence that surgical intervention with telescopic rod is an effective way for femoral fractures or deformities in children with OI.

## Supplementary Information

**Additional file 1:** **Supplementary Fig 1.** AP femur radiographs of the plate and screws (a-c), the telescopic rod (d-f), the non-elongating rod (g-i) and the elastic nail (j-m). The preoperative radiographs (a, d, g, j) and postoperative radiographs (b, e, h, k) of the plate group, the telescopic nail group, the non-elongating rod group and the elastic nail group were shown respectively. Refracture happened in two ends of the plate (c), refracture happened in proximal femur (f), refracture happened in bony segment without supporting (i) and refracture owing to insufficient supporting (j) were the main complications for revision surgery in each group.

## Data Availability

The datasets used and analysed during the current study are available from the corresponding author on reasonable request.

## Abbreviations

OI: Osteogenesis imperfecta
IQR: Interquartile range
CI: Confidence interval
