# Supplementary material for: Which is the best femoral implant in children with osteogenesis imperfecta? a retrospective cohort study of 783 procedures
Source: BMC Musculoskelet Disord. 2023 Feb 9;24:110. doi: 10.1186/s12891-023-06222-2 (PMC9909911; doi:10.1186/s12891-023-06222-2)
Supplement: Supplementary file 1 — Additional file 1: Supplementary Fig 1. AP femur radiographs of the plate and screws (a-c), the telescopic rod (d-f), the non-elongating rod (g-i) and the elastic nail (j-m). The preoperative radiographs (a, d, g, j) and postoperative radiographs (b, e, h, k) of the plate group, the telescopic nail group, the non-elongating rod group and the elastic nail group were shown respectively. Refracture happened in two ends of the plate (c), refracture happened in proximal femur (f), refracture happened in bony segment without supporting (i) and refracture owing to insufficient supporting (j) were the main complications for revision surgery in each group. [file 12891_2023_6222_MOESM1_ESM.docx]

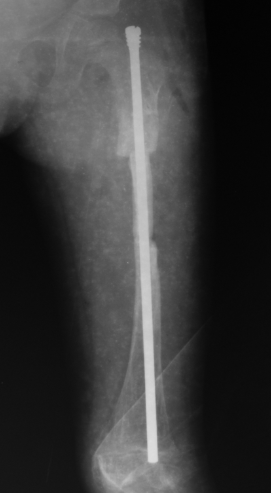

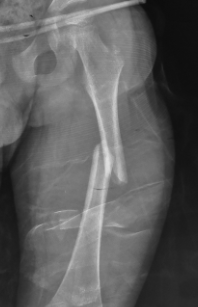

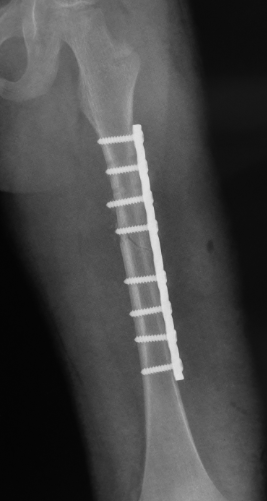

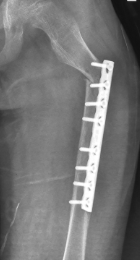

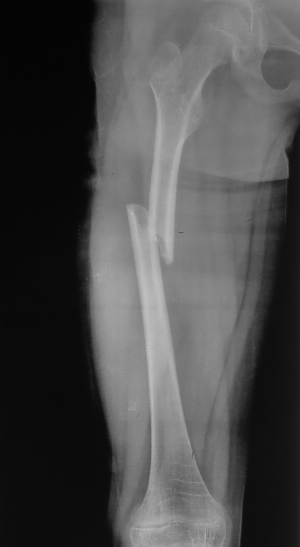

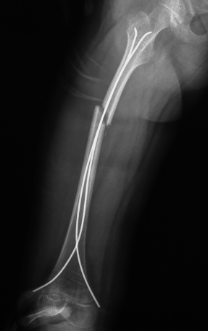

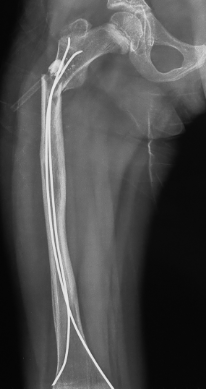

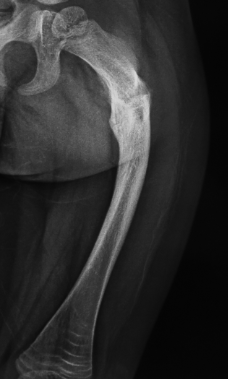

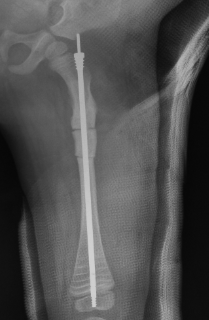

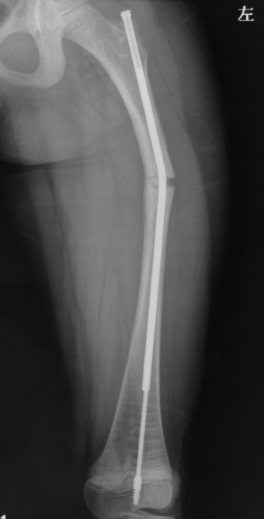

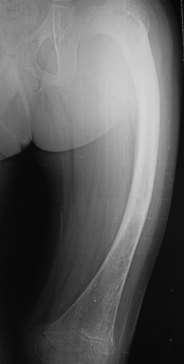

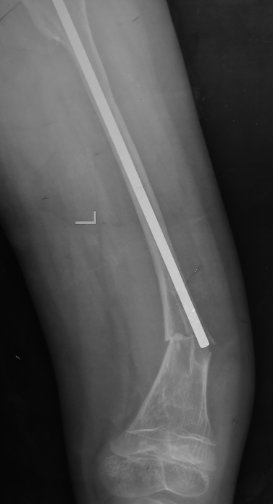


a

b

c

d

e

f

g

m

k

j

i

h

**Supplementary figure 1**

**Supplementary Fig. 1** AP femur radiographs of the plate and screws (a-c), the telescopic rod (d-f), the non-elongating rod (g-i) and the elastic nail (j-m). The preoperative radiographs (a, d, g, j) and postoperative radiographs (b, e, h, k) of the plate group, the telescopic nail group, the non-elongating rod group and the elastic nail group were shown respectively. Refracture happened in two ends of the plate (c), refracture happened in proximal femur (f), refracture happened in bony segment without supporting (i) and refracture owing to insufficient supporting (j) were the main complications for revision surgery in each group.
